# Risk factors for mortality in infants with congenital diaphragmatic hernia: a single center experience

**DOI:** 10.1007/s00508-021-01843-w

**Published:** 2021-03-30

**Authors:** Jennifer Bettina Brandt, Tobias Werther, Erika Groth, Erik Küng, Johann Golej, Angelika Berger

**Affiliations:** 1 Comprehensive Centre for Paediatrics, Division of Neonatology, Paediatric Intensive Care & Neuropaediatrics, Department of Paediatric and Adolescent Medicine, Medical University of Vienna, Waehringer Guertel 18–20, 1090 Vienna, Austria; 2 Department of General Medicine, Glasgow Royal Infirmary, Glasgow, UK

**Keywords:** CDH, Pulmonary hypertension, ECMO, Inhaled nitric oxide, Survival parameters

## Abstract

**Background:**

Despite current progress in research of congenital diaphragmatic hernia, its management remains challenging, requiring an interdisciplinary team for optimal treatment.

**Objective:**

Aim of the present study was to evaluate potential risk factors for mortality of infants with congenital diaphragmatic hernia.

**Methods:**

A single-center chart review of all patients treated with congenital diaphragmatic hernia over a period of 16 years, at the Medical University of Vienna, was performed. A comparison of medical parameters between survivors and non-survivors, as well as to published literature was conducted.

**Results:**

During the observational period 66 patients were diagnosed with congenital diaphragmatic hernia. Overall survival was 84.6%. Left-sided hernia occurred in 51 patients (78.5%) with a mortality of 7.8%. In comparison, right-sided hernia occurred less frequently (*n* = 12) but showed a higher mortality (33.3%, *p* = 0.000). Critically instable patients were provided with venoarterial extracorporeal membrane oxygenation (ECMO, 32.3%, *n* = 21). Survival rate among these patients was 66.7%. Right-sided hernia, treatment with inhaled nitric oxide (iNO) over 15 days and the use of ECMO over 10 days were significant risk factors for mortality.

**Conclusion:**

The survival rate in this cohort is comparable to the current literature. Parameters such as the side of the diaphragmatic defect, duration of ECMO and inhaled nitric oxide were assessed as mortality risk factors. This analysis of patients with congenital diaphragmatic hernia enhances understanding of risk factors for mortality, helping to improve management and enabling further evaluation in prospective clinical trials.

## Introduction

Congenital diaphragmatic hernia (CDH) occurs in approximately 1 in 2500–5000 live births [1, 2]. Research in animal models has enhanced knowledge of its pathogenesis, involving developmental abnormalities of the pleuroperitoneal fold [3]. Neonates with CDH often present with additional malformations and chromosomal aberrations [4]. Management of these patients is a demanding challenge and requires a multidisciplinary team [5]. Mortality rates of CDH range from 52% to 82%, varying among pediatric centers and depending on various factors and comorbidities [6, 7, 8]. Different risk factors including side of defect, position of liver, necessity of extracorporeal membrane oxygenation (ECMO), pulmonary hypertension (PH) and additional congenital anomalies contribute to the high mortality [2, 9, 10].

The aim of this study was to perform a retrospective chart review of CDH in a single tertiary pediatric center, to analyze risk factors for mortality and to compare the results with the current literature.

## Patients and methods

This study was conducted as a single center analysis at the Medical University of Vienna. We performed a retrospective chart review of all infants diagnosed with CDH and treated at our neonatal or pediatric intensive care unit (ICU) between 2000 and 2015, to analyze risk factors for mortality and to compare results with current literature. All participants were born either in this center or another Austrian hospital and were transferred to our department before CDH repair was performed. Primary outcome was survival until discharge from the ICU. We created a retrospective registry including the following demographic and medical parameters: gender, gestational age (GA), birth weight (BW), side of defect, position of liver, length of stay in ICU, presence of additional congenital comorbidities, PH including treatment with inhaled nitric oxide (iNO), timing of surgical intervention, operational technique and surgical complications, as well as necessity and length of ECMO treatment (Medtronic Biomedicus 560 centrifugal pump, Dublin, Ireland).

Descriptive statistics were presented as mean and standard deviation (SD) or range for continuous variables and as absolute and relative frequencies for categorical variables. A data comparison was performed between survivors and non-survivors (until discharge from or death in the ICU). For determination of statistical significance in categorical variables a χ^2^-test was used, while continuous variables calculations were conducted by using the two-tailed unpaired *t*-test. Statistical significance was considered to be achieved with a *p*-value < 0.05. The *p*-values were not adjusted for multiple testing and have to be interpreted as explorative only. For variables with a *p*-value < 0.05 in the univariate analysis, relative risk was calculated, using a 2×2 table with Haldane correction as appropriate, unconditional maximum likelihood estimation for confidence intervals and a mid‑P exact test for significance. Univariate analysis and data visualization were performed using the R statistic environment with the ggplot2 package [11, 12]. Data collection was performed in Excel© (Microsoft Cooperation, Albuquerque, New Mexico, United States, Version 15.19.1).

## Results

Between 2000 and 2015 a total of 66 patients with CDH were treated in our center. One patient born at 29 weeks GA with 900 g BW, who died on the first day of life due to refractory arterial hypotension was excluded from analyses. Overall survival in our cohort was 84.6%. Comparison of demographic and medical data between survivors (*n* = 55) and non-survivors (*n* = 10) is presented in Table 1. One survivor was treated in our ICU for 133 days. This patient was transferred to a German CDH center, while still on the ventilator, at parental request.

**Table 1:** Characteristics of survivors and non-survivors

Characteristics	Survivors (*n* = 55)	Non-survivors (*n* = 10)	*p*-value
Female, *n*	24 (43.6)	5 (50)	0.71
Gestational age at birth, weeks (SD)	37.6 (1.9)	37.2 (2.3)	0.48
Birth weight, g (SD)	2949 (570)	2406 (541)	0.56
Vaginal delivery, *n* ^a^	14 (25.5)	0 (0)	0.09
Out-born, *n*	20 (36.4)	4 (40)	0.83
Length of stay in ICU, days (SD)	16.9 (23.6)	17.7 (14.2)	0.31
*Anomalies*			
L‑CDH, *n*	47 (85.5)	4 (40)	< 0.001
Intrathoracic liver, *n* ^b^	28 (50.9)	8 (80)	0.04
Comorbidities, *n*	16 (29.1)	3 (30)	1.00
Chromosomal aberrations, *n*	2 (3.6)	0 (0)	0.83
Cardiac defects, *n*	9 (16.4)	2 (20)	0.67
Other comorbidities, *n*	5 (9.1)	1 (10)	1.00
*PH and ventilation*			
Ventilation, days (SD)	17.4 (22.4)	17.7 (14.2)	0.19
Pulmonary hypertension, *n*	37 (67.3)	10 (100)	0.03
iNO, *n*	37 (67.3)	10 (100)	0.03
Duration of iNO treatment, days (SD)	4.5 (6.8)	14.7 (14.3)	0.02
*ECMO*			
VA-ECMO, *n*	14 (25.5)	7 (70)	0.01
ECMO treatment, days (SD)	2.4 (4.4)	9.3 (11.1)	0.01
*Surgery*			
Surgical intervention, *n* ^c^	55 (100)	7 (70)	0.00
Timing of surgery, days (SD)	9.1 (26.6)	10.1 (9)	0.05
Patch, *n*	18 (32.7)	5 (50)	0.05
Surgical complications, *n*	8 (14.5)	2 (20)	0.31
Need of reoperation, *n*	5 (9.1)	2 (20)	0.18

Data are presented as numbers (%) and mean (SD) unless otherwise indicated. *P*-values were calculated using the χ^2^-testor the two-tailed unpaired t‑test

*n* number, *SD* standard deviation, *ICU* intensive care unit, *L‑CDH* left sided congenital diaphragmatic hernia, *PH* pulmonary hypertension, *iNO* inhalative nitric oxide, *VA-ECMO* venoarterial extracorporeal membrane oxygenation

^a^ In 5 patients, mode of delivery could not be determined due to lack of documentation

^b^ For 2 patients no information about the position of liver was available

^c^ In 3 patients no operation was performed. Two patients died due to treatment refractory cardiorespiratory failure (one of them on ECMO) and one patient died on ECMO after severe cerebral hemorrhage, before any surgical intervention was performed

There was no significant difference between survivors and non-survivors with respect to gender, GA, BW, mode of delivery or place of birth (Table 1). Survival of in-born patients was not higher than of out-born patients (*n* = 35, 85.4% vs. *n* = 20, 83.3%, *p* = 0.83). The majority of out-born patients, however, underwent surgical repair by stitch (87.5% vs. 47.4% in the in-born cohort, *p* = 0.001).

Table 2 gives detailed information about patients with left-sided CDH (L-CDH) and right-sided CDH (R-CDH). Of all patients 51 (78.5%) were diagnosed with L‑CDH and 12 patients (18.5%) with R‑CDH. Mortality in patients with R‑CDH was higher compared to patients with L‑CDH (33.3% versus 7.8%, *p* = 0.02). Two patients (3%) had defects on both sides; both did not survive (one had a complete aplasia of the diaphragm and died due to bleeding on ECMO and the other patient could not be hemodynamically stabilized despite ECMO treatment).

**Table 2:** Characteristics of patients with left-sided versus right-sided congenital diaphragmatic hernia

	L‑CDH (*n* = 51)	R‑CDH (*n* = 12)	*p*-value
Survivors, *n*	47 (92.2)	8 (66.7)	0.02
Intrathoracic liver, *n* ^a^	22 (43.1)	12 (100)	0.002
Intra-abdominal liver, *n* ^a^	27 (52.9)	0 (0)	0.002
Comorbidities, *n*	13 (25.5)	5 (41.7)	0.30
Cardiac defect, *n*	7 (13.7)	4 (33.3)	0.20
Pulmonary hypertension, *n*	35 (68.6)	10 (83.3)	0.31
iNO, *n*	35 (68.6)	10 (83.3)	0.31
Days of ventilation, mean (SD) ^b^	15.5 (15.6)	27.3 (35.6)	0.02
ECMO, *n* ^c^	15 (29.4)	5 (41.7)	0.50
Patch repair, *n* ^d^	16 (31.4)	7 (58.3)	0.09
Stitch repair, *n* ^d^	34 (66.7)	5 (41.7)	0.09

Data are presented as numbers (percentage) and mean (SD) unless otherwise indicated. *P*-values were calculated using the χ^2^-testor the two-tailed unpaired t‑test

*n* number, *SD* standard deviation, *L‑CDH* left sided congenital diaphragmatic hernia, *R‑CDH* right sided congenital diaphragmatic hernia, *ECMO* extracorporeal membrane oxygenation

^a^ In 2 patients the position of liver could not be determined; both of them had L‑CDH

^b^ In 4 patients duration of ventilation was missing

^c^ One patient on ECMO had bilateral CDH

^d^ In 3 patients no intervention could be performed, 2 of them had bilateral CDH

Intrathoracic position of the liver was found in 50.9% of survivors as opposed to 80% of non-survivors (*p* = 0.04). Two patient charts did not contain any information about the position of the liver; both had L‑CDH. All patients with R‑CDH (*n* = 12) and bilateral hernia (*n* = 2) had intrathoracic parts of the liver, as opposed to 22 patients (43.1%) with L‑CDH (*p* = 0.01, Table 2). Out of 36 patients 8 (22.2%) with intrathoracic position of the liver died as opposed to only 1 of 27 (3.7%) patients with an entirely intra-abdominal position of the liver (*p* = 0.04).

Of the patients 19 (29.2%) had documented comorbidities, 8 patients had various anomalies, including chromosomal anomalies (*n* = 2), esophageal atresia (*n* = 1), dysmorphic syndrome (*n* = 1), fetofetal transfusion syndrome (*n* = 1), bilateral hydronephrosis with hydroureter (*n* = 1), a congenital cervical tumor (*n* = 1), and a congenital cystic adenomatoid malformation (*n* = 1). The other 11 patients (57.9%) had cardiac malformations: atrial septal defect (*n* = 8), stenosis of the pulmonary artery (*n* = 1), pulmonary atresia (*n* = 1) and aortic isthmus stenosis (*n* = 1). A cardiac defect was found in 4 out of 12 patients with R‑CDH (33.3%, 50% mortality) and 7 out of 51 patients with L‑CDH (13.7%, 0% mortality, Table 2).

**Fig. 1:**
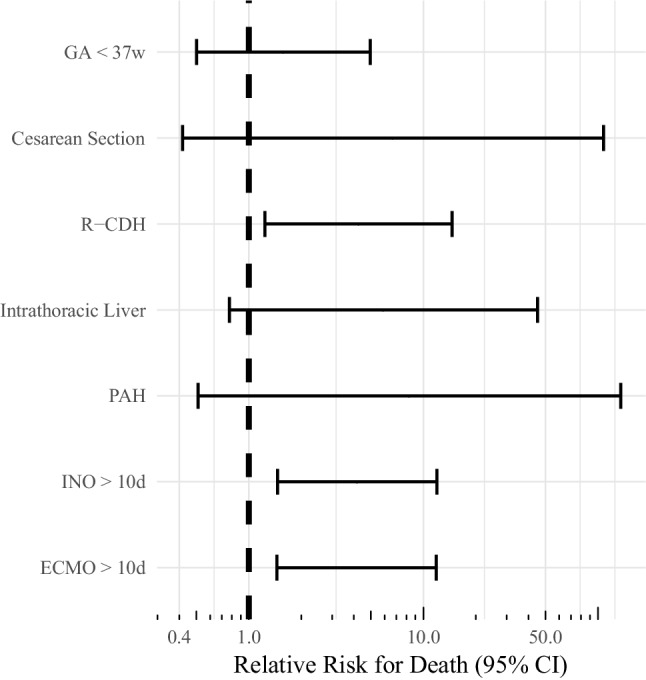
Relative risk for death in children with congenital diaphragmatic hernia. *GA* *<* *37w* gestational age below 37 weeks; *R‑CDH* right-sided congenital diaphragmatic hernia, *PAH* pulmonary hypertension; *INO* *>* *10d* inhalative nitric oxide over 10 days; *ECMO* *>* *10d* extracorporeal membrane oxygenation over 10 days, *CI* confidence interval

Pulmonary hypertension was diagnosed in 47 patients (72.3%). Infants suffering from PH showed a longer period of mechanical ventilation (21.5 vs. 6.3 mean days, *p* = 0.02). All 18 patients without PH survived, whereas mortality was 21.3% in patients with PH (*n* = 10, *p* = 0.03) and 20 patients on ECMO (95.2%) had PH. All patients diagnosed with PH received iNO. In 29 patients iNO treatment was continued after surgical repair. Mortality among these patients was 24.1% (*n* = 7). Mean length of iNO treatment was 6.3 days (SD 9.3 days, longest duration 42 days), with a longer duration in non-survivors (mean 14.7 days, SD 14.3 days) compared to survivors (mean 4.5 days, SD 6.8 days, *p* = 0.02). Administration of iNO for more than 10 days was associated with a fourfold increase of mortality (Fig. 1).

Of all patients 21 (32.3%) received VA ECMO with an overall mortality of 33.3% (*n* = 7, Table 3) as opposed to 6.8% (*n* = 3) of patients without ECMO (*p* = 0.01). Of these patients 2 could not be hemodynamically stabilized and died on ECMO prior to any surgical intervention, ECMO was initiated after a mean of 2.2 days of life (SD = 3.2 days), 8 patients were on ECMO only prior to surgery and could successfully be decannulated either before or immediately after the intervention (mean = 9 days, SD = 2.3, 0% mortality), 9 patients were still in need of ECMO after surgery (mean = 4 days, SD = 2.5, 55.6% mortality) and 2 patients needed ECMO only after surgery (for 9 and 14 days, respectively). Mean length of ECMO treatment overall was 10.8 days (2–36 days, SD 6.6 days). The longest duration of ECMO treatment among survivors was 14 days. Patients receiving ECMO for more than 10 days (*n* = 10) had a mortality of 40%, compared to 27.3% in patients receiving ECMO for less than 10 days (*n* = 11, *p* = 0.29). The relative risk for death was four times higher in patients on ECMO for more than 10 days (Fig. 1).

**Table 3:** Characteristics of patients on ECMO versus no ECMO

Characteristics	ECMO (*n* = 21)	No ECMO (*n* = 44)	*p*-value
Mortality, *n*	7 (33.3)	3 (6.8)	0.01
Gestational age at birth, weeks (SD)	37.3 (2.0)	37.6 (2.0)	0.13
Birth weight, g, weight (SD)	2802.5 (510)	2896 (634.6)	0.56
Length of stay in ICU, days (SD)	31.8 (32)	9.9 (10.4)	0.05
Intrathoracic liver, *n* ^a^	15 (71.4)	21 (47.7)	0.05
R‑CDH, *n*	5 (23.8)	7 (15.9)	0.62
Comorbidities, *n*	6 (28.6)	13 (29.5)	1.00
Cardiac defect, *n*	4 (19)	7 (15.9)	0.74
Duration of ventilation, days (SD)	29.7 (31.3)	11.1 (8.1)	0.02
Pulmonary hypertension, *n*	20 (95.2)	27 (61.4)	0.004
Duration of iNO treatment, days (SD)	11.2 (10.5)	3.7 (7.5)	0.11
Timing of surgery, days of life (SD) ^b^	9.2 (6.2)	9.3 (30.1)	0.001
Patch repair, *n* ^b^	11 (52.4)	12 (27.3)	0.02
Surgical complications, *n* ^b^	5 (23.8)	5 (11.4)	0.26

Data are reported as numbers (percentage) and mean [SD] unless otherwise indicated. *P*-values were calculated using the χ^2^-testor the two-tailed unpaired t‑test

*n* number, *SD* standard deviation, *g* grams, *ICU* intensive care unit, *L‑CDH* left sided congenital diaphragmatic hernia, *R‑CDH* right sided congenital diaphragmatic hernia, *PH* pulmonary hypertension, *iNO* inhalative nitric oxide, *ECMO* extracorporeal membrane oxygenation

^a^ In 2 patients the position of liver was unknown

^b^ Two patients on ECMO had no surgical intervention

Of all patients 62 (95.4%) underwent surgery, 3 patients (4.8%) died before any surgical intervention was performed, 2 of them died on ECMO. Two different methods (patch for larger sized defects or stitch for smaller sized defects) were conducted to repair the diaphragm. Stitch repair was the most common method (*n* = 39, 62.9%). Patch repair was performed in 23 patients (37.1%). Patients with patch-closed hernia showed a trend to increased mortality in comparison to stitch-closed CDH (patch mortality 21.7% vs. stitch mortality 5.1%, *p* = 0.05). Out of 19 patients on ECMO 11 (57.9%) had a patch repair of the diaphragmatic defect. In contrast, 72.1% of patients repaired by stitch were not on ECMO (*n* = 31, *p* = 0.03).

## Discussion

In this single-center 16-year experience of 65 patients with congenital diaphragmatic hernia, we report an overall survival of 84.6%. These results are within the upper range of the current literature [7, 8, 13–20]. Right-sided CDH, treatment with iNO > 15 days and the use of ECMO > 10 days were significant risk factors for mortality in our cohort. We did not find a significant effect of GA and BW on survival in accordance with Hoffman et al. who showed that BW was not a predictor for mortality in patients on ECMO [21], and Colvin et al. who found no effect of GA on mortality either [6]. Delaplain et al. reported a higher risk of mortality for patients with lower BW [22]. Also, Kadir et al. indicated that risk of mortality declined by 7% for every 100 g increase in BW [8]. Survival of in-born patients was not higher than of out-born patients in our cohort. A possible explanation for this finding could be that out-born infants with severe CDH might have died before transfer, which could not be considered in our analyses due to lack of documentation. In fact, the majority of out-born and transferred patients underwent surgical repair by stitch indicating a less severe and smaller defect of the diaphragm. Our data support the observation that L‑CDH occurs more often than R‑CDH [3–5] and that overall mortality in infants with R‑CDH is higher than in infants with L‑CDH [4, 22]. Difficulty of prenatal diagnosis [10], necessity of patch repair [23, 24] as well as reoperation are possible reasons for higher mortality among patients with R‑CDH [23]. In contrast to data reported by Duess et al. [9] our findings showed no higher demand for ECMO among patients with R‑CDH. Intrathoracic localization of the liver has repeatedly been reported to be associated with increased mortality, as also found in our cohort, probably due to distinctive pulmonary hypoplasia [2, 14, 17]. Similarly, our data showed a trend towards increased mortality in patients with patch repair in comparison to stitch repair, as also described in other reports [9, 18, 24].

Presence of additional comorbidities is known to limit survival [25]. Published trials showed that patients with additional cardiac anomalies have lower chances of survival [23, 26]. In accordance with published data [5, 26], cardiac anomalies were the most frequently documented additional defects in our cohort, although we found no effect on mortality as opposed to Graziano et al. [27]. This discrepancy could be explained by the difference in sample size (65 patients in our cohort as opposed to more than 2600 patients in the study of Graziano et al.) and the exclusion of atrial septal defects in the study of Graziano et al. due to the lack of hemodynamic significance, which were, however, the most common defects in our cohort. Our data support the observation that survival with extracorporeal support as rescue therapy in severe CDH is lower compared to patients without requirement of ECMO treatment [6]; however, it has been shown that application of ECMO increases survival of patients who are unresponsive to conventional treatment [21, 23]. Morini et al. demonstrated that mortality rates declined from 83.5% to 38.3% when ECMO treatment was applied in patients unresponsive to conventional treatment [28]. In our cohort, 66.7% of CDH infants receiving ECMO survived. This percentage is in the upper range of reports in the literature, ranging from 50–75% [3, 9, 15, 19, 23, 30]. In accordance to published data [29], we observed decreased survival in infants with prolonged need of ECMO treatment. Our data showed a fourfold increase in relative risk of death in patients with ECMO treatment exceeding 10 days, which supports the findings presented by McHoney et al. [5] Pulmonary hypertension has been described as a major risk factor for mortality in patients with CDH [1, 3]. Our analysis also showed increased mortality in patients with a diagnosis of PH as well as longer periods of ventilation, treatment with iNO and necessity of ECMO.

Limitations of this study are its retrospective design and the long time period covered, resulting in incomplete availability of data and individual parameters. On the other hand, given the long time period considered, we were able to report a sufficiently high number of patients to draw conclusions from a single-center experience.

In conclusion, our data on basic characteristics, management and outcome of patients with CDH over a 16-year period in a single tertiary referral center, contribute to the comprehension of predictors for mortality of patients with this rare condition and might help to improve future management of patients with CDH as well as the design of prospective studies.

## Abbreviations

BW: Birth weight
CDH: Congenital diaphragmatic hernia
GA: Gestational age
ICU: Intensive care unit
iNO: Inhaled nitric oxide
L‑CDH: Left-sided CDH
PH: Pulmonary hypertension
R‑CDH: Right-sided CDH
VA ECMO: Venoarterial extracorporeal membrane oxygenation
